# Abdominal Wall Endometrioma after Laparoscopic Operation of Uterine Endometriosis

**DOI:** 10.1155/2016/5843179

**Published:** 2016-05-31

**Authors:** Tihomir Vukšić, Pejana Rastović, Vedran Dragišić

**Affiliations:** Department of Surgery, University Clinical Hospital Mostar, Bijeli Brijeg b.b., 88000 Mostar, Bosnia and Herzegovina

## Abstract

Endometriosis is presence of functional endometrium outside of uterine cavum. As a pluripotent tissue, endometrium has the possibility of implanting itself almost everywhere; even implantation in abdominal wall was described, but it is not common site. This case report presents implantation of functional endometrium in abdominal wall, inside scar tissue, and after insertion of a laparoscopic trocar port. Final diagnosis was confirmed by pathohistological examination.

## 1. Introduction

Endometriosis is defined as a presence of functional endometrium outside of uterine cavum and is usually localized in the pelvic cavity. Cause of this disorder is still insufficiently known, and the most popular theory is reflux of endometrium and its implantation, mostly on the organs and peritoneum of the pelvic cavity. Reflux theory cannot explain presence of endometriosis inside of parenchymatous organs as the lungs, brain, or gastrointestinal wall. Other popular theories are immunologic alteration, pluripotent celom epithelium alteration, and the differentiation of progenitor stem cell [1]. Presence of implanted endometrium inside of scar tissue after obstetric or surgical operations, or traumas, mostly in form of tumor-endometrioma, is described in several case reports, and it suggests that implantation is unpredictable and not usual after wound contamination with endometrium. Leite et al. in their work from 2009 estimate that endometrioma will appear in 0.03 to 3.5% of cases after obstetric procedure [2]. Average age of patients with endometrioma is 31.4 years. 96% of patients present with a tumor, 87% had pain, and 57% had symptoms related to menstrual cycles. Average time of appearance of endometrioma after initial operation is 3.6 years. Surgical resection leads to healing in 95% of cases, and recidives appear in 4.3% of cases [3]. After introduction of laparoscopic procedures in diagnosis and treatment of endometriosis, certain cases of implanted endometrium in trocar port wound appeared, and until now 15 of similar cases are reported [4]. Polish author Chmaj-Wierzchowska after studying this phenomenon, as well as related literature, claims that implantation of endometriosis in scars after laparoscopic procedure appears in 0.5–7% of cases [5].

## 2. Case Report

Forty-three-year-old patient is admitted to the surgical clinic because of a tumor localized on inferior left side of abdominal wall. Two years before patient underwent laparoscopic surgery for uterine endometriosis. After several months, patient noticed the tumor in scar area of trocar port, which had been growing during this time. Tumor did not cause any discomfort, it was painless on palpation, and it did not change during the menstrual cycles. After surgical examination, the possibility of endometrial implantation is considered, and radical excision of tumor is indicated. Irregularly shaped tumor about 4 cm in diameter, which had infiltrated the complete thickness of the abdominal wall including muscles and fascia, had been found. The complete tumor had been removed and sent to pathohistological examination. Postoperative recovery went without complications, with the wound healing by primary intention.

## 3. Pathohistological Finding

**Figure 1:**
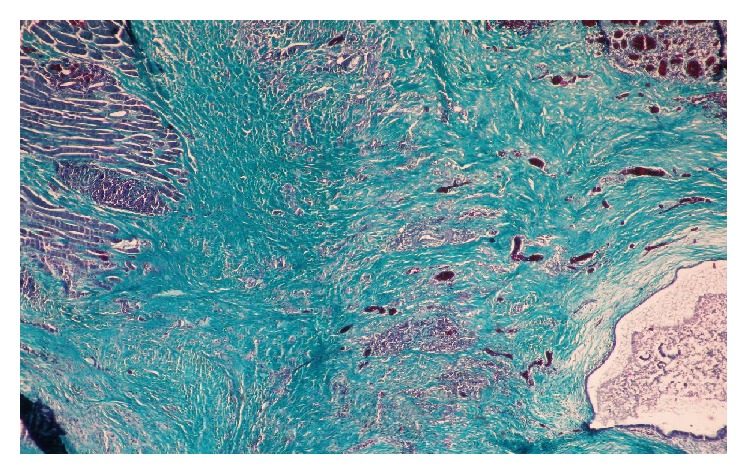
Mallory, 4x. On right side skeletal muscle tissue is visible, and on left side area of endometrial stroma and glands can be seen.

Material is a slice of tissue measuring 3.5 cm on the longer axis, haemorrhagic on section. Upon pathohistologic examination fat and connective tissue was seen as well as parts of muscle tissue with endometrial glands surrounded with endometrial stroma and filled with blood. Histological finding primarily fits the diagnosis of endometriosis (Figure 1).

## 4. Discussion

First case report about endometriosis inside of a scar after laparoscopic procedure was reported by the American author Healy in 1995. He had logically predicted that similar cases would appear with increasing use of laparoscopy in diagnostic and operative procedures. He had also stressed the importance of general surgeons in treatment of such findings [6]. Patients with endometriosis inside of a scar after recent surgical procedure are mostly referred to a general surgeon because of the open etiology of the tumor mass, which includes incision hernias, granuloma, or haematoma [7]. During the removal of endometrioma, the general surgical rule of requiring wide excision of tumor to healthy tissue is considered valid. With such approach we are removing tissue that is not examined pathohistologically yet, and we are not sure of its oncological character. In cases of endometrioma, wide excision prevents possible recidive [4, 8]. Endometriosis alternates into malignant form in about 1% of cases, mostly altering to clear cell carcinoma, and malignant alternation of abdominal wall scar endometrioma is described. The unique case of atypical endometriosis which altered to carcinosarcoma has also been described. For the surgeon this is one reason more for wide excision of endometrioma [9, 10]. From those case reports, it is clear that implantation of pluripotent endometrium is iatrogenic and that operator must maintain proper surgical technique to prevent or at least reduce the appearance of endometrioma inside of a scar. Proper surgical technique includes usage of Endobag, irrigation of the incision wound, and careful suturing. Elevated postoperative levels of CA-125 markers can be a useful sign of endometriosis recidive, although there are no papers connecting abdominal wall endometrioma and the above-mentioned marker, especially after laparoscopic procedures [11]. Substantial evidence goes in favor of using oral contraceptive after laparoscopic operations of endometriosis to prevent its recidive [12].

## 5. Conclusion

Recidive of endometriosis in scar after recent operation, especially laparoscopic one, is not a common finding and because of that it is often overlooked. In patients with palpable tumor on site of the surgical scar, which does not have to be changed with menstrual cycles, and with recent surgically treated endometriosis, possibility of recidive of endometriosis in form of endometrioma has to be considered. Listed data and clinical findings are sufficient for proper diagnosis. Preoperative conformation can be obtained by fine needle aspiration biopsy of tumor.
